# *Trypanosoma* spp. infection in urban and wild ecotopes of the caribbean region in Colombia

**DOI:** 10.17843/rpmesp.2024.412.13598

**Published:** 2024-05-16

**Authors:** Iván Benavides-Céspedes, Marlon Mauricio Ardila, Geovanny Jiménez-Cotes, Luis Avendaño-Maldonado, Daisy Lozano-Arias, Roberto Garcia-Alzate, Leidi Herrera

**Affiliations:** 1 Basic Sciences Faculty, Universidad del Atlántico, Puerto Colombia, Colombia. Universidad del Atlántico Basic Sciences Faculty Universidad del Atlántico Puerto Colombia Colombia; 2 Departamento de Patología y Medicina Preventiva, Facultad de Ciencias Veterinarias, Universidad de Concepción, Chillán, Chile. Universidad de Concepción Departamento de Patología y Medicina Preventiva Facultad de Ciencias Veterinarias Universidad de Concepción Chillán Chile; 3 Basic and Clinical Research Group in Health Sciences, Health Sciences Faculty, Fundación Universitaria San Martín, Puerto Colombia, Colombia. Basic and Clinical Research Group in Health Sciences Health Sciences Faculty Fundación Universitaria San Martín Puerto Colombia Colombia; 4 Center for Ecology and Evolution, Institute of Zoology and Tropical Ecology (IZET), Faculty of Sciences, Universidad Central de Venezuela, Caracas, Venezuela. Universidad Central de Venezuela Center for Ecology and Evolution, Institute of Zoology and Tropical Ecology Faculty of Sciences Universidad Central de Venezuela Caracas Venezuela; 5 Instituto en Ciencias de la Salud, Universidad Nacional de Asunción, Paraguay. Universidad Nacional de Asunción Instituto en Ciencias de la Salud Universidad Nacional de Asunción Paraguay

**Keywords:** Colombia, bats, Trypanosoma, Zoonoses

## Abstract

We conducted a study to evaluate the frequency of infection by *Trypanosoma* spp. in bats captured in wild and urban ecotopes in the Department of Atlántico in the Caribbean region of Colombia from March 2021 to May 2022. Bats were taxonomically identified, and sex, relative age, and reproductive conditions were determined. A blood sample was used for parasitological analysis and DNA extraction to amplify a region of the 18S rRNA. 125 bats were collected, with the most abundant families being *Molossidae* (62/125; 49.6%) and *Phyllostomidae* (43/125; 34.4%). *Molossus molossus* collected in wild habitats showed an infection frequency of 8.1% (5/61) and 4.1% (3/61) through parasitological and molecular analysis, respectively. In comparison, *Noctilio albiventris* collected in urban habitats showed an infection frequency of 16.6% (2/12) for both analyses. These findings represent the first records of *M. molossus* harboring trypanosomes for the Department of Atlántico and of *N. albiventris* harboring trypanosomes in Colombia.

## INTRODUCTION

Bats are hosts and reservoirs of several parasitic microorganisms ^1^. Their ability to fly, longevity, mobility, and ecosystem services as seed dispersers, pollinators, and arthropod controllers place bats in the focus of eco-epidemiological surveillance for some zoonoses ^2^. Some etiological agents of these zoonoses, such as trypanosomatids, including *Trypanosoma* and *Leishmania* (Euglenozoa: Kinetoplastea, *Trypanosomatidae*), are important in veterinary and human medicine ^1^^,^^3^.

*Trypanosoma cruzi*, a parasite that causes Chagas disease (also known as American trypanosomiasis), affects people in Colombia. By 2023, there were 34 reported cases of acute Chagas disease ^4^. The way this disease spreads (transmission cycles) changes depending on the animals living in each area (ecotope). This shift is seen with *T. cruzi*and other similar parasites (trypanosomes). In some regions, the types of mammals that feed the disease-carrying insects (vectors) may influence the severity (virulence) and prevalence of the parasite ^5^.

It has been investigated since 1982 that bats in Colombia may carry the Trypanosoma parasite. At that time, Marinkelle found *T. cruzi* in 233 bat blood samples, *Trypanosoma cruzi marinkellei*in 25 samples, and other *Schizotrypanum* species in 315 samples ^6^. These infections were more common in central, eastern, and southern Colombia. Although bats from the Caribbean region were included in those studies, the frequency of Trypanosoma infection was not reported. That is why this study was designed to evaluate how frequently wild and urban bats in the Department of Atlántico carry Trypanosoma species and to understand their potential in the transmission of Chagas disease.

**KEY MESSAGES**

Motivation for the study. The role of bats as hosts of *Trypanosoma* spp. in the Atlantic department in Colombia, as well as its taxonomic diversity has been poorly studied.

Main findings. This is the first report of frequency of infection by *Trypanosoma* spp. in bats in the Atlántico Department in Colombia.

Implications. The great adaptive capacity of bats to different ecological niches and its role as hosts of *Trypanosoma* spp. for wild and urban ecotopes represents a risk factor in transmission cycles of epidemiological importance.

## THE STUDY

### Study area

**Figure 1:**
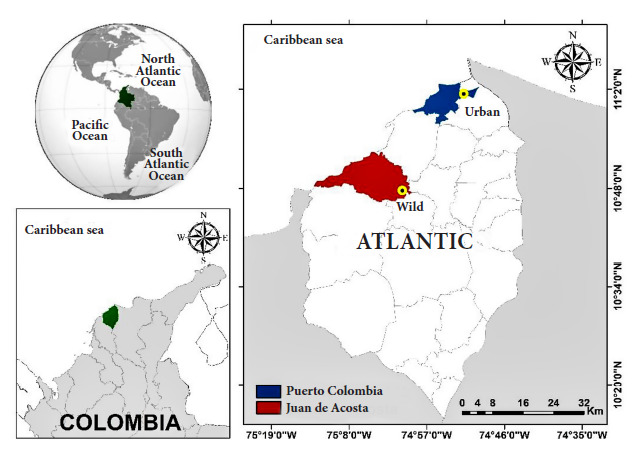
Study area of the collection of bats in wild (red) and urban (blue) ecotopes of the Atlántico Department, Colombian Caribbean region.

The sampling area consisted of four collection points, two in urban ecotopes delimited as point 1U (11º01'02" N-74º52'30" W) and point 2U (11º01'00" N-74º52'28" W), within 895.6 ha of tropical dry forest on the Campus of the Universidad del Atlántico in the Puerto Colombia Municipality (average elevation: 21 meters), with an average temperature between 28°-32° C and an annual rainfall of 819 mm^3^, and two in wild ecotopes delimited as point 1W (10º47'54" N - 75º00'35" W) and point 2W (10º47'53" N - 75º00'34" W) formed within 1,425.2 ha of tropical dry forest in the Corregimiento of Chorrera (Juan de Acosta Municipality) (average elevation: 70 meters), average temperature between 27°-32° C and annual rainfall of 1,655 mm^3^, all from the Atlántico Department, northern of Colombia ^7^ (Figure 1).

### Capture, identification and marking of bats

We used two mist nets (12x2.5 meters, 3x3 cm mesh; BioWed®) to capture bats from March 2021 to May 2022. In both wild and urban areas, we conducted three sampling sessions over two consecutive nights (between 5:30 pm and 11:30 pm), totaling six sessions (12 nights) ^8^. The total effort was 4,320 m2 per hour, with an effort per night of 180 hours per network.

The taxonomic identification of the specimens was carried out with the keys of Díaz*et al.*^9^. Sex, age, and reproductive condition were determined following methods in Kunz*et al*. ^10^. Microperforations were made in the left wing patagium to register recaptured. Another volume of blood was destined for parasitological analysis. The morphometric data for each animal were recorded on a self-prepared information sheet.

Parasitological and molecular diagnosis

Sterile blood (50 µL) was collected from the brachial vein. For subsequent molecular analysis, the blood was impregnated with Whatman filter paper and stored at 4°C. Each fresh blood sample was examined twice under an optical microscope (Leica Microsystems CMS GmbH, model DMi1, Morrisville, NC, USA) at 40X to search for haemoflagellates morphologically compatible with trypanosoma and their possible quantification ^(^^11^.

We used the Wizard® HMW purification kit (Promega) to extract DNA from blood samples soaked in filter paper, following the manufacturer's instructions. We then quantified the total DNA concentration using an EPOCH 2NS instrument (BioTeck Instruments). To detect *Trypanosoma* spp., we performed a nested PCR (Polymerase Chain Reaction) targeting a specific region of the 18S rRNA gene (positions 200-600). In the first PCR step, we used the primers TRY-927 forward and TRY-927 reverse. Positive samples generated a fragment of about 900 base pairs (bp) ^12^. For the second PCR step, 5 µL of the first PCR product was used with the primers SSU-561 forward and SSU-561 reverse, amplifying a fragment between 320 and 560 bp ^12^. To ensure the test's accuracy, we included a negative control (PCR mix without DNA) and a positive control (*T. cruzi*DNA, strain MDID/CO/2018/Dm006).

The PCR mix was carried out with a final volume of 25 µL, of which 5 µL corresponded to the template DNA and the remaining 20 µL to 10 µL of GoTaq® Green Master Mix, 2X (Promega), 3 µL of MgCl2, 2 µL of each of the primers implemented for each stage and 5 μl of nuclease-free water (Promega®). PCR was carried out in the TC-9639 thermocycler (Benchmark SCIENTIFIC: Sayreville, NJ, USA) following the conditions of Noyes *et al.*^12^. The products obtained were evidenced by horizontal electrophoresis (100V/30 minutes) in 1.5% agarose gel, stained with Ethidium Bromide solution in TAE buffer, for 12 minutes, to be visualized and photo documented in the iBright™ imaging system FL1500 (Thermo Fisher Scientific Inc: MA, USA).

Samples were considered positive when a band between 320-560 bp was amplified ^12^, using the 100-1,000 bp molecular size marker (MBiotech) as a reference. 

### Data analysis

The infection rate was estimated as a percentage, and the comparative analysis of the diversity of the number of bat species per location (q0/species richness; q1/typical species, and q2/dominant species) was carried out based on abundance data according to Hill ^13^. For this analysis, species rarefaction curves (Hill numbers) were performed using the "iNext" package in the R language ^14^. A permutation test was performed in the PAST-4 program to look for significant differences between the diversity of order q0, q1, and q2 of bats captured in wild and urban locations. A Fisher's exact test was used to determine the association between the presence of Trypanosoma spp. in bats and ecotopes, and a chi-square test was used to determine the statistical differences between the infection rate in the collected bat species/ecotope.

### Ethical aspects

The study was approved by the Ethics Committee of the Universidad del Atlántico, Puerto Colombia, Colombia, with the code 02-III-2021.

## FINDINGS

### Richness, abundance and morphological characteristics of bats

A total of 125 bats were captured, belonging to four families and eight species, which were distributed in four trophic guilds ^15^. The most abundant families were*Molossidae*(62/125, 49.6%) and*Phyllostomidae*(43/125, 34.4%), followed by*Noctilionidae*(18/125, 14.4%) and*Vespertilionidae*(2/125, 1.6%).

**Figure 2:**
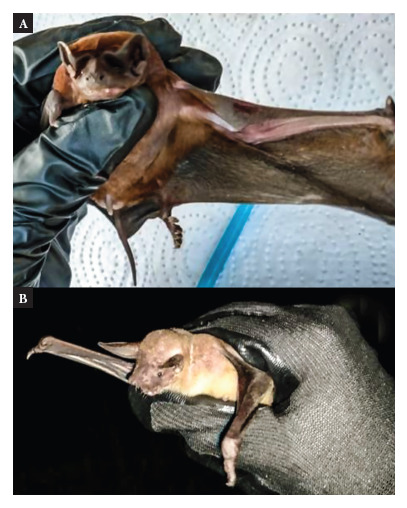
Bats captured in the Atlántico Department, Caribbean region of Colombia. A. Species *Molossus molossus*, B. Species *Noctilio albiventris.* Source: Grupo Interdisciplinario en Ciencias Marinas y Ambientales (GICMARA), Universidad del Atlántico.

Within the general external morphological characteristics of the bats studied, individuals of*Molossus molossus*(Figure 2A) exhibit a bicolor coat that is 4 mm in length, starting with a light base and transitioning to a reddish-brown. Additionally, the hair at the hip´s base measures 7.3 mm. These bats have an approximate weight of 18,8 g, an average forearm length of 40.1 mm, and a wingspan of 288.1 mm ^15^. In addition,*Noctilio albiventris*individuals (Figure 2B) feature short, reddish-yellow fur, weighs 26.7 g, and have a forearm length of 59,3 mm and a wingspan of 421.6 mm ^15^.

The species by area, the trophic guild, sex, relative age, and reproductive conditions to which they belong were complemented in Table 1.

**Table 1:** Characteristics of bats captured in the Atlántico Department, Colombian Caribbean region.

Ecotope	Family	Species	Trophic guild	Reproductive status										Age M				Age F				Total Number of individuals
				M.sa	M.sna	Total	F.sa	F.sna	F.p	F.l	F.pl	F.cc	Total	A	SA	J	Total	A	SA	J	Total	
Wild	*Molossidae*	*Molossus molossus*	Insectivorous	22	2	24	25	10	1	2	0	0	38	20	2	2	24	28	3	7	38	62
	*Phyllostomidae*	*Artibeus jamaicensis*	Mainly frugivorous	9	0	9	1	0	0	0	4	0	5	9	0	0	9	5	0	0	5	14
		*Glossophaga soricina*	Mainly nectarivorous	5	0	5	1	0	0	0	0	0	1	4	1	0	5	1	0	0	1	6
		*Artibeus lituratus*	Mainly frugivorous	0	0	0	1	0	0	0	0	0	1	0	0	0	0	1	0	0	1	1
		*Uroderma convexum*	Mainly frugivorous	1	0	1	0	0	0	0	0	0	0	1	0	0	1	0	0	0	0	1
	*Noctilionidae*	*Noctilio leporinus*	Mainly piscivorous	1	0	1	0	0	1	0	0	0	1	1	0	0	1	1	0	0	1	2
	*Vespertilionidae*	*Myotis* sp*.*	Insectivorous	0	0	0	1	1	0	0	0	0	2	0	0	0	0	1	0	1	2	2
	Total			38	2	40	29	11	2	2	4	0	48	35	3	2	40	37	3	8	48	88
Urban	*Phyllostomidae*	*Artibeus jamaicensis*	Mainly frugivorous	8	0	8	3	2	1	2	1	0	9	8	0	0	8	7	1	1	9	17
		*Glossophaga soricina*	Mainly nectarivorous	3	0	3	1	0	0	0	0	0	1	3	0	0	3	1	0	0	1	4
	*Noctilionidae*	*Noctilio albiventris*	Mainly insectivorous	4	1	5	4	2	4	1	0	0	11	4	0	1	5	9	1	1	11	16
	Total			15	1	16	8	4	5	3	1	0	21	15	0	1	16	17	2	2	21	37

M: Male; F: Female; M.sa: Sexually active male; M.sna: Sexually non-active male; F.sa: Sexually active female; F.sna: Sexually non-active female; F.p: Pregnant female; F.l: Lactating female; F.pl: Post-lactation female; F.cc: Female with calf; A: Adult; SA: Subadult; J: Juvenile; T: Total.

**Figure 3:**
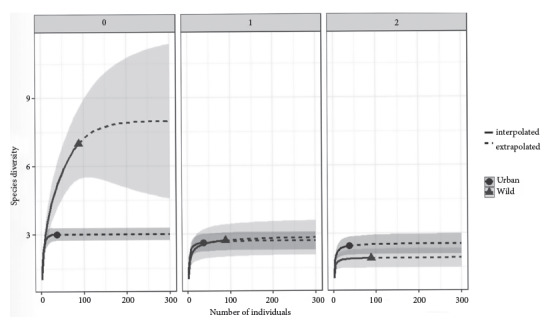
Rarefaction curve for diversity of order q0, q1, and q2 of assemblages of bats collected in wild and urban ecotopes of the Atlántico Department, Colombian Caribbean region.

The alpha diversity indices (Hill numbers) revealed a richness q0 for both areas, with the wild area presenting significantly higher richness than the urban area (p=0.010), even though the curve of rarefaction for the wild area is not stable. The values of q1 for both zones revealed similar abundances but without significant differences (p=0.080), and both rarefaction curves tend to stabilize, a trend that is repeated for the values of q2, where it was observed a greater dominance in the assemblage of wild bats concerning the urban one, but without significant differences (p=0.080) (Figure 3).

### Frequency of infection by trypanosoma spp. in bats

**Figure 4:**
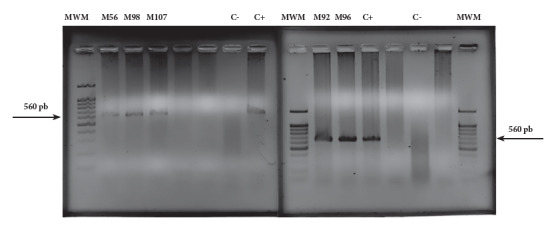
Visualization of the nested PCR products of the blood samples extracted from bats captured in wild and urban ecotopes of the Atlántico Department (Colombian Caribbean region) in 1.5% agarose gel. MWM: molecular weight marker (100-1,000 bp); M56, M92, M96, M98, M107: total DNA samples extracted from blood soaked in filter paper with their respective codes; C+: positive control (*Trypanosoma cruzi* DNA strain MDID/CO/2018/Dm006); C-: negative control.

Of the 125 captured bats, seven pregnant females were excluded (two from the wild ecotope and five from the urban one), and 94.4% (118/125) of the bats were analyzed. Regarding the molecular diagnosis, it was obtained that 4.2% (5/118) of the samples amplified a band of 560 bp (Figure 4). In detail,*M. molossus*from the wild ecotope was found positive with 2.5% (3/118), while in the urban ecotope*N. albiventris*with 1.6% (2/118).

The frequency of infection by*Trypanosoma*spp. according to parasitological analyses for bats captured in wild ecotopes, it was 5.8% (5/86), and for urban ecotopes it was 6.2% (2/32). The bat species with the presence of sanguineous trypomastigotes were*M. molossus*(for wild ecotopes) and*N. albiventris*(for urban ecotopes). The parasitological diagnosis for*M. molossus*was 8.1% (5/61), regarding the molecular (4.9%, 3/61)*. N. albiventris*presented the same frequency of infection through parasitological and molecular diagnosis (16.6%, 2/12).

No association was found between the presence of*Trypanosoma*spp. in bats and the ecotope (Fischer; p=1.000), as well as no significant differences between the frequency of infection by these parasites in bats from wild areas (2.5%) and those from urban areas (1.6%) (p=0.929).

## DISCUSSION

Few studies have dealt with the biodiversity of the bat fauna of the Atlántico Department, and this is the first to contribute to updating the inventory of bats inhabiting assemblages of wild and urban ecotopes with tropical dry forest remnants, also including a first approximation of the presence of *Trypanosoma* spp. in these mammals. The present study covered 57.1% of the families reported in the Atlántico Department ^16^ and 44.4% of those recorded in the Neotropics ^17^.

When looking at the total number of bat species (q0), our data suggests a higher potential species richness in the wild habitat compared to the urban habitat. This indicates greater bat diversity in wild areas. The stable rarefaction curves for both wild and urban areas (q1) suggest our sampling effort was sufficient to capture a good representation of the most abundant bat species in each location. Interestingly, even though the urban area has fewer bat species overall, the distribution of those species appears to be more even (q2). However, this difference between wild and urban areas is not statistically significant (p=0.080).

The role of *M. molossus* as a trypanosomatids host has been reported in Venezuela by Añez *et al*. ^11^, with evidence of congenital transmission of *T. cruzi*, and in Brazil by Oliveira da Silva ^17^ with 54.0% of infection frequency (7/13) for *T. cruzi* and 69.0% (9/13) for *Leishmania* spp. For Colombia, the role of *M. molossus* as a host for trypanosomatids has been studied in the region of Vichada, with an infection frequency of 83.3% (10/12) for *T. cruzi*^1^, and in the region of Casanare, with the first report of *L. amazonensis* in this species, without data on infection frequency ^3^; in turn, the same author analyzed blood samples from six bats from the Atlántico Department, without specifying the species, but noting the absence of trypanosomatids infections. Thus, the present study would be the first report of the species *M. molossus* as a host for *Trypanosoma* spp. in the Atlántico Department.

The presence of T. cruzi-TcBat in *N. albiventris* from Brazil was reported by Lima *et al*. ^18^. Marinkelle ^6^ reported infection with *Schizotrypanum* sp. in *N. labialis*, a synonym of *N. leporinus*, using parasitological techniques (50.0%, 157/315); this synonym was established as a species by Solari *et al*. ^19^. The present results would correspond to the first record of *N. albiventris* as a host of *Trypanosoma* spp. in Colombia. It was not possible to obtain the surrounding *Trypanosoma* species by genomic sequencing in the present study due to resource limitations.

In conclusion, the results suggest that the presence of bats infected with *Trypanosoma* spp. (with observed blood trypomastigotes) represents a potential risk, as they may come into contact with biological vectors, with the possibility of causing zoonotic diseases. Future studies will be necessary to identify the *Trypanosoma* species in bats and their role in the epidemiological scenario as a reservoir of trypanosomatids. This study contributes to strengthening the inventories of bat fauna in the municipalities of the Atlántico Department in the Caribbean region of Colombia.
