# Supplementary material for: Trypanosoma spp. infection in urban and wild ecotopes of the caribbean region in Colombia
Source: Rev Peru Med Exp Salud Publica. 2024 May 16;41(2):156–63. doi: 10.17843/rpmesp.2024.412.13598 (PMC11300683; doi:10.17843/rpmesp.2024.412.13598)
Supplement: Translation

# Translation: Infección por *Trypanosoma* spp. en murciélagos capturados en ecótopos urbanos y silvestres de la región Caribe en Colombia

**Authors:** Iván Benavides-Céspedes, Marlon Mauricio Ardila, Geovanny Jiménez-Cotes, Luis Avendaño-Maldonado, Daisy Lozano-Arias, Roberto Garcia-Alzate, Leidi Herrera

**Affiliations:** 1 Facultad de Ciencias Básicas, Universidad del Atlántico, Puerto Colombia, Colombia; 2 Departamento de Patología y Medicina Preventiva, Facultad de Ciencias Veterinarias, Universidad de Concepción, Chillán, Chile; 3 Grupo de Investigación Básica y Clínica en Ciencias de la Salud, Facultad de Ciencias de la Salud, Fundación Universitaria San Martín, Puerto Colombia, Colombia.; 4 Centro de Ecología y Evolución, Instituto de Zoología y Ecología Tropical, Facultad de Ciencias, Universidad Central de Venezuela, Caracas, Venezuela.; 5 Instituto en Ciencias de la Salud, Universidad Nacional de Asunción, Paraguay.

**Keywords:** Colombia, Murciélagos, Trypanosoma, Zoonosis

## Abstract

Se evaluó la frecuencia de infección por *Trypanosoma* spp. en murciélagos capturados en ecótopos silvestres y urbanos del Departamento del Atlántico, en la región Caribe de Colombia, entre marzo de 2021 y mayo de 2022. Se identificaron taxonómicamente los murciélagos y se determinó sexo, edad relativa y condiciones reproductivas. Se utilizó una muestra de sangre para análisis parasitológico y extracción de ADN para la amplificar una región del ARNr 18S. Se capturaron 125 murciélagos, siendo las familias más abundantes *Molossidae* (62/125; 49,6%) y Phyllostomidae (43/125; 34,4%). *Molossus molossu*s capturado en ecótopos silvestres mostró una frecuencia de infección del 8,1% (5/61) y 4,1% (3/61) mediante análisis parasitológico y molecular, respectivamente. En comparación, *Noctilio albiventris* capturado en ecótopos urbanos mostró una frecuencia de infección del 16,6% (2/12) para ambos análisis. Estos hallazgos representan los primeros registros de *M. molossus* albergando *Trypanosoma* spp. para el Departamento del Atlántico y de *N. albiventris* albergando *Trypanosoma* spp. en Colombia.

## INTRODUCCIÓN

Los murciélagos son hospederos y reservorios de varios microorganismos parásitos ^1^. Su capacidad para volar, longevidad, movilidad y servicios ecosistémicos como dispersores de semillas, polinizadores y controladores de artrópodos sitúan a los murciélagos en el foco de la vigilancia ecoepidemiológica de algunas zoonosis ^2^. Algunos agentes etiológicos de estas zoonosis, como los tripanosomátidos, entre ellos *Trypanosoma* y *Leishmania* (Euglenozoa: Kinetoplastea, *Trypanosomatidae*), son importantes en medicina veterinaria y humana ^1^^,^^3^.

*Trypanosoma (Schizotrypanum) cruzi,* es el agente causal de la Tripanosomiasis Americana o enfermedad de Chagas, y afecta a los colombianos siendo reportados 34 casos para el año 2023 ^4^. La transición de la enzootia estricta a la zooantroponosis, condicionada por la fauna presente en cada ecótopo, se reflejan en los ciclos de transmisión de *T. cruzi* y otros tripanosomátidos. En algunas regiones, el mantenimiento del aporte sanguíneo a los vectores a través de diferentes mamíferos podría modular la virulencia de los parásitos y su prevalencia ^5^.

Los estudios sobre murciélagos como hospederos de *Trypanosoma* en Colombia se remontan a 1982, cuando Marinkelle ^6^ reportó 233 hemocultivos positivos para *T. cruzi*, 25 para *T. cruzi marinkellei* y 315 para *Schizotrypanum* spp. La mayor prevalencia se encontró en las regiones central, oriental y sur de Colombia. Aunque los estudios incluyeron murciélagos de la región Caribe, no se reportó la frecuencia de infección por *Trypanosoma*. Por lo tanto, el objetivo de este estudio fue evaluar la frecuencia de infección por *Trypanosoma* spp. en murciélagos silvestres y urbanos del Departamento del Atlántico en Colombia, para conocer su posible rol en los ciclos epidemiológicos las Triponosomiasis.

**MENSAJES CLAVE**

Motivación para realizar el estudio. El rol de los murciélagos como hospederos de *Trypanosoma* spp. en el Departamento del Atlántico en Colombia, así como su diversidad taxonómica ha sido poco estudiada.

Principales hallazgos. Este es el primer reporte de frecuencia de infección por *Trypanosoma* spp. en murciélagos en el Departamento del Atlántico en Colombia.

Implicaciones. La gran capacidad de adaptación de los murciélagos a diferentes nichos ecológicos y su rol como hospederos de *Trypanosoma* spp. en ecótopos silvestres y urbanos representa un factor de riesgo en ciclos de transmisión de importancia epidemiológica.

## EL ESTUDIO

### Área de estudio

El área de muestreo consistió en cuatro puntos de colecta, dos en ecótopos urbanos delimitados como punto 1U (11º01’02” N-74º52’30” O) y punto 2U (11º01’00” N-74º52’28” O), dentro de 895,6 hectáreas de Bosque Seco Tropical (BsT) en el Campus de la Universidad del Atlántico en el Municipio de Puerto Colombia (elevación promedio: 21 metros), con una temperatura promedio entre 28°-32° C y una precipitación anual de 819 mm^3^, y dos en ecótopos silvestres delimitados como punto 1W (10º47’54” N - 75º00’35” O) y punto 2W (10º47’53” N - 75º00’34” O) conformados dentro de 1425,2 hectáreas de bosque seco tropical BsT en el Corregimiento de Chorrera (Municipio de Juan de Acosta) (elevación promedio: 70 metros), temperatura promedio entre 27° a 32° C y precipitación anual de 1655 mm^3^, todos del Departamento del Atlántico, norte de Colombia ^(^^7^ (Figura 1).

**Figura 1:** Área de recolección de murciélagos en ecótopos silvestres (rojo) y urbanos (azul) del Departamento del Atlántico, región del Caribe Colombiana.

### Captura, identificación, y marcaje de murciélagos

Se utilizaron dos redes de niebla de 12 x 2,5 m y un tamaño de malla de 3 x 3 cm (BioWed®) para capturar murciélagos entre marzo de 2021 y mayo de 2022. Se realizaron tres sesiones de muestreo en dos noches consecutivas (entre las 17:30 y 23:30 horas) en zonas silvestres y urbanas, sumando seis sesiones de muestreo (12 noches) ^8^*.* El esfuerzo total fue de 4320 m^2^ por hora, con un esfuerzo por noche de 180 horas por red.

La identificación taxonómica de los ejemplares se realizó con las claves de Díaz *et al.*^9^, así como la determinación del sexo, edad y condiciones reproductivas se ajustaron a Kunz *et al.*^10^. Se realizaron microperforaciones en el patagio del ala izquierda para registrar las recapturas. Otro volumen de sangre se destinó al análisis parasitológico. Los datos morfométricos de cada animal se registraron en una ficha informativa de elaboración propia.

### Diagnóstico parasitológico y molecular

Se extrajo sangre estéril (50 µL) de la vena braquial. Para el posterior análisis molecular, la sangre se impregnó con papel de filtro Whatman y se almacenó a 4 °C. Cada muestra de sangre fresca se examinó dos veces con un microscopio óptico (Leica Microsystems CMS GmbH, modelo DMi1, Morrisville, NC, EE. UU.) a 40X para buscar hemoflagelados, morfológicamente compatibles con el *Trypanosoma*, y su posible cuantificación ^(^^11^.

La extracción del ADN de las muestras de sangre impregnada en papel de filtro se realizó con el kit de purificación Wizard® HMW (Promega Corporation: Madison, WI, EE.UU.), siguiendo las instrucciones del fabricante. La concentración del ADN total se cuantificó utilizando un EPOCH 2NS (BioTeck Instruments, Inc: EE.UU.), y el diagnóstico molecular se realizó mediante amplificación por Reacción en Cadena de la Polimerasa (PCR) anidada de una región comprendida entre las posiciones 200 y 600 de la fracción 18S del ARNr, para determinar la presencia de *Trypanosoma* spp, utilizando los cebadores TRY-927 Forward (5’GAAACAAGAAACACGGAG3’) y TRY-927 Reverse (5’CTACTGGGCAGCTTGGA3’), que en los casos positivos generaron un fragmento de aproximadamente 900 pb ^12^. Posteriormente, se tomaron 5 µL del producto de amplificación y se realizó una segunda etapa de PCR, utilizando los cebadores SSU-561 *Forward* (5’TGGGATAACAAAGGAGCA3’) y SSU-561 *Reverse* (5’CTGAGACTGTAACCTCAAAGC3’) que amplificaron un fragmento entre 320 y 560 pb ^12^. Se utilizó una mezcla de PCR sin ADN como control negativo de la reacción, y ADN de *T. cruzi* (cepa MDID/CO/2018/Dm006) como control positivo.

La mezcla de PCR se realizó con un volumen final de 25 µL, de los cuales 5 µL correspondieron al ADN molde y los 20 µL restantes a 10 µL de GoTaq® Green Master Mix, 2X (Promega), 3 µL de MgCl_2_, 2 µL de cada uno de los cebadores implementados para cada etapa y 5 μL de agua libre de nucleasas (Promega®). La PCR se llevó a cabo en el termociclador TC-9639 (Benchmark SCIENTIFIC: Sayreville, NJ, EE.UU.) siguiendo las condiciones de Noyes *et al.*^12^. Los productos obtenidos se evidenciaron mediante electroforesis horizontal (100V/30 minutos) en gel de agarosa al 1,5%, teñido con solución de bromuro de etidio en tampón TAE, durante 12 minutos, para ser visualizados y fotodocumentados en el sistema de imagen iBright™ FL1500 (Thermo Fisher Scientific Inc: MA, EE.UU.). Las muestras se consideraron positivas cuando se amplificó una banda entre 320-560 pb ^12^, utilizando como referencia el marcador de tamaño molecular de 100-1.000 pb (MBiotech).

### Análisis de datos

La frecuencia de infección se estimó en porcentajes y el análisis comparativo de la diversidad del número de especies de murciélagos por localidad (q0/riqueza de especies; q1/especie típica y q2/especie dominante) se realizó a partir de los datos de abundancia según Hill ^13^. Para este análisis, se realizaron curvas de rarefacción de especies (números de Hill) utilizando el paquete “iNext” del lenguaje R ^14^.

Se realizó una prueba de permutación en el programa PAST-4 para buscar diferencias significativas entre la diversidad de orden q0, q1 y q2 de los murciélagos capturados en localidad silvestre y urbana. Una prueba de Fisher permitió determinar la asociación entre la presencia de *Trypanosoma* spp. en los murciélagos y los ecótopos y una prueba de chi-cuadrado permitió determinar las diferencias estadísticas entre la frecuencia de infección en las especies de murciélagos/ecótopos colectados.

### Aspectos éticos

El estudio fue aprobado por el Comité de Ética de la Universidad del Atlántico, Puerto Colombia, Colombia, con el código 02-III-2021.

## HALLAZGOS

### Riqueza, abundancia y características morfológicas de los murciélagos

Se capturaron 125 murciélagos, pertenecientes a cuatro familias y ocho especies, que se distribuyeron en cuatro gremios tróficos ^15^. Las familias más abundantes fueron *Molossidae* (62/125; 49,6%) y *Phyllostomidae* (43/125; 34,4%), seguidas de *Noctilionidae* (18/125; 14,4%) y *Vespertilionidae* (2/125; 1,6%). Dentro de las características morfológicas externas generales de los murciélagos estudiados, los individuos de *Molossus molossus* (Figura 2A) presentan un pelaje bicolor de 4 mm de longitud, que comienza con una base clara y pasa a un marrón rojizo. Además, el pelo de la base de la cadera mide 7,3 mm. Estos murciélagos tienen un peso aproximado de 18,8 g, una longitud media del antebrazo de 40,1 mm y una envergadura alar de 288,1 mm ^15^. Además, los individuos de *Noctilio albiventris* (Figura 2B) presentan un pelaje corto de color amarillo rojizo, pesan 26,7 g y tienen una longitud de antebrazo de 59,3 mm y una envergadura de 421,6 mm ^15^.

**Figura 2:** Murciélagos capturados en el Departamento del Atlántico, región Caribe de Colombia. A. Especie Molossus molossus, B. Especie Noctilio albiventris. Fuente. Grupo Interdisciplinario en Ciencias Marinas y Ambientales (GICMARA), Facultad deFacultad de Ciencias Básicas, Universidad del Atlántico.

Las especies por área, gremio trófico, sexo, edad relativa y condiciones reproductivas a las que pertenecen se complementaron en la Tabla 1. Los índices de diversidad alfa (números de Hill) revelaron una riqueza de especies (q0) para ambas áreas, con el área silvestre presentando una riqueza significativamente mayor que el área urbana (p=0,010), aunque la curva de rarefacción para el área silvestre no es estable. Los valores de q1 (riqueza de especies) para ambas zonas revelaron abundancias similares, pero sin diferencias significativas (p=0,080), y ambas curvas de rarefacción tienden a estabilizarse, tendencia que se repite para los valores de q2 (especie típica), donde se observó una dominancia de los murciélagos silvestres con respecto al urbano, pero sin diferencias significativas (p=0,080) (Figura 3).

**Tabla 1:** Características de los murciélagos capturados en el Departamento del Atlántico, región del Caribe de Colombia.

Ecótopo	Familia	Especies	Gremio trófico	Estado reproductivo										Edad M				Edad H				Número total de individuos
				M.sa	M.sna	T	H.sa	H.sna	H.p	H.l	H.pl	H.cc	T	A	SA	J	T	A	SA	J	T	
Silvestre	*Molossidae*	*Molossus molossus*	Insectívoro	22	2	24	25	10	1	2	0	0	38	20	2	2	24	28	3	7	38	62
	*Phyllostomidae*	*Artibeus jamaicensis*	Principalmente frugívoro	9	0	9	1	0	0	0	4	0	5	9	0	0	9	5	0	0	5	14
		*Glossophaga soricina*	Principalmente nectarívoro	5	0	5	1	0	0	0	0	0	1	4	1	0	5	1	0	0	1	6
		*Artibeus lituratus*	Principalmente frugívoro	0	0	0	1	0	0	0	0	0	1	0	0	0	0	1	0	0	1	1
		*Uroderma convexum*	Principalmente frugívoro	1	0	1	0	0	0	0	0	0	0	1	0	0	1	0	0	0	0	1
	*Noctilionidae*	*Noctilio leporinus*	Principalmente piscívoro	1	0	1	0	0	1	0	0	0	1	1	0	0	1	1	0	0	1	2
	*Vespertilionidae*	*Myotis* sp*.*	Insectívoro	0	0	0	1	1	0	0	0	0	2	0	0	0	0	1	0	1	2	2
	Total			38	2	40	29	11	2	2	4	0	48	35	3	2	40	37	3	8	48	88
Urbano	*Phyllostomidae*	*Artibeus jamaicensis*	Principalmente frugívoro	8	0	8	3	2	1	2	1	0	9	8	0	0	8	7	1	1	9	17
		*Glossophaga soricina*	Principalmente nectarívoro	3	0	3	1	0	0	0	0	0	1	3	0	0	3	1	0	0	1	4
	*Noctilionidae*	*Noctilio albiventris*	Principalmente insectívoro	4	1	5	4	2	4	1	0	0	11	4	0	1	5	9	1	1	11	16
	Total			15	1	16	8	4	5	3	1	0	21	15	0	1	16	17	2	2	21	37

M: Macho; H: Hembra; M.sa: Macho sexualmente activo; M.sna: Macho sexualmente no activo; H.sa: Hembra sexualmente activa; H.sna: Hembra sexualmente no activa; H.p: Hembra preñada; H.l: Hembra lactante; H.pl: Hembra postlactante; H.cc: Hembra con cría; A: Adulto; SA: Subadulto; J: Juvenil; T: Total.

**Figura 3:** Curva de rarefacción para la diversidad de orden q=0 (riqueza de especies), q=1 (especie típica) y q=2 (especie dominante) de ensamblajes de murciélagos capturados en ecótopos silvestres y urbanos del Departamento del Atlántico, región del Caribe de Colombia

### Frecuencia de infección por *Trypanosoma* spp. en murciélagos

De los 125 murciélagos capturados, siete hembras preñadas fueron excluidas (dos del ecótopo silvestre y cinco del urbano), el 94,4% (118/125) de los murciélagos fueron analizados. En cuanto al diagnóstico molecular, se obtuvo que el 4,2% (5/118) de las muestras amplificaron una banda de 560 bp (Figura 4). En detalle, *M. molossus* del ecótopo silvestre se encontró positivo con 2,5% (3/118), mientras que en el ecótopo urbano se encontró a *N. albiventris* con 1,6% (2/118).

**Figura 4:** Visualización de los productos de PCR anidada de las muestras de sangre de murciélagos capturados en ecótopos silvestres y urbanos del Departamento del Atlántico, región Caribe de Colombia en gel de agarosa al 1,5%. MPM: marcador de tamaño molecular (100-1.000 pb); M56, M92, M96, M98, M107: muestras de ADN total extraídas de sangre impregnada en papel filtro con sus respectivos códigos; C+: control positivo (ADN de Trypanosoma cruzi cepa MDID/CO/2018/Dm006); C-: control negativo.

La frecuencia de infección por *Trypanosoma* spp. de acuerdo con los análisis parasitológicos para los murciélagos capturados en ecótopos silvestres fue del 5,8% (5/86) y para los ecótopos urbanos fue del 6,2% (2/32). Las especies de murciélagos con presencia de tripomastigotes sanguíneos fueron *M. molossus* (para ecótopos silvestres) y *N. albiventris* (para ecotó pos urbanos).

El diagnóstico parasitológico de *M. molossus* fue del 8,1% (5/61), con respecto al molecular (4,9%, 3/61). *N. albiventris* presentó la misma frecuencia de infección por diagnóstico parasitológico y molecular (16,6%, 2/12).

No se encontró asociación entre la presencia de *Trypanosoma* spp. en murciélagos y en los ecótopos (Fischer; p=1,000), así como no hay diferencias significativas entre la frecuencia de infección por estos parásitos en murciélagos de áreas silvestres (2,5%) y los de áreas urbanas (1,6%) (p= 0,929).

## DISCUSIÓN

Pocos estudios han abordado la biodiversidad de la fauna de murciélagos del Departamento del Atlántico en la región Caribe de Colombia, y este es el primero en contribuir a la actualización del inventario de murciélagos que habitan en ensamblajes de ecótopos silvestres y urbanos con remanentes de Bst, incluyendo también una primera aproximación de la presencia de *Trypanosoma* spp. en estos mamíferos. El presente estudio abarcó el 57,1% de las familias reportadas en el Departamento del Atlántico ^16^ y el 44,4% de las registradas en el Neotrópico ^(^^17^.

En cuanto a la estructura comunitaria con mayor esfuerzo de muestreo (q0), se pudieron conocer más especies en el ensamblaje de la zona silvestre, que presentaba una mayor diversidad de murciélagos con respecto a la zona urbana. Las curvas de rarefacción más estables para las áreas silvestres y urbanas (q1) reflejan un buen esfuerzo de captura que proporciona una contribución aceptable al inventario de especies basado en abundancias. Aunque el área urbana tiene una menor riqueza de murciélagos, este ensamblaje tiene una mejor uniformidad (q2), aunque no hay diferencias significativas entre las áreas.

El rol de *M. molossus* como hospedero de tripanosomátidos ha sido reportado en Venezuela por Añez *et al.*^11^, con evidencia de transmisión congénita de *T. cruzi*, y en Brasil por Oliveira da Silva ^17^ con 54,0% de frecuencia de infección (7/13) para *T. cruzi* y 69,0% (9/13) para *Leishmania* spp. En Colombia, se ha estudiado el papel de *M. molossus* como hospedero de tripanosomátidos en el Departamento de Vichada, con una frecuencia de infección del 83,3% (10/12) para *T. cruzi*^1^, y en el Departamento de Casanare, con el primer reporte de *L. amazonensis* en esta especie, sin datos sobre la frecuencia de infección ^3^; a su vez, el mismo autor analizó muestras de sangre de seis murciélagos del Departamento del Atlántico, sin especificar la especie, pero observando la ausencia de infecciones por tripanosomátidos. Así, el presente estudio sería el primer reporte de la especie *M. molossus* como hospedero de *Trypanosoma* spp. en el Departamento del Atlántico en Colombia.

La presencia de *T. cruzi*-TcBat, en *N. albiventris* de Brasil fue reportada por Lima *et al.*^18^. Marinkelle ^6^ reportó infección por *Schizotrypanum* sp. en *N. labialis*, sinónimo de *N. leporinus* por técnicas parasitológicas (50,0%, 157/315); este sinónimo fue establecido como especie por Solari *et al.*^19^. Los presentes resultados corresponderían al primer registro de *N. albiventris* como hospedero de *Trypanosoma* spp. en Colombia. No fue posible obtener las especies de *Trypanosoma* circundantes por secuenciación genómica en el presente estudio debido a la limitación de recursos.

En conclusión, los resultados sugieren que la presencia de murciélagos infectados con *Trypanosoma* spp. (observando tripomastigotes sanguíneos) representa un riesgo potencial, ya que pueden entrar en contacto con vectores biológicos, con la posibilidad de causar zoonosis. Serán necesarios estudios futuros para identificar las especies de *Trypanosoma* en los murciélagos y su rol en el escenario epidemiológico como reservorio de tripanosomátidos. Este estudio contribuye al fortalecimiento de los inventarios de la fauna de murciélagos en los municipios del Departamento del Atlántico en la región Caribe de Colombia.
